# Characterisation of InGaN by Photoconductive Atomic Force Microscopy

**DOI:** 10.3390/ma11101794

**Published:** 2018-09-21

**Authors:** Thomas F. K. Weatherley, Fabien C.-P. Massabuau, Menno J. Kappers, Rachel A. Oliver

**Affiliations:** Department of Materials Science & Metallurgy, University of Cambridge, 27 Charles Babbage Road, Cambridge CB3 0FS, UK; thomas.weatherley@epfl.ch (T.F.K.W.); fm350@cam.ac.uk (F.C.-P.M.); mjk30@cam.ac.uk (M.J.K.)

**Keywords:** InGaN, photoconductive atomic force microscopy, dislocations

## Abstract

Nanoscale structure has a large effect on the optoelectronic properties of InGaN, a material vital for energy saving technologies such as light emitting diodes. Photoconductive atomic force microscopy (PC-AFM) provides a new way to investigate this effect. In this study, PC-AFM was used to characterise four thick (∼130 nm) In${}_{x}$Ga${}_{1-x}$N films with *x* = 5%, 9%, 12%, and 15%. Lower photocurrent was observed on elevated ridges around defects (such as V-pits) in the films with x≤12%. Current-voltage curve analysis using the PC-AFM setup showed that this was due to a higher turn-on voltage on these ridges compared to surrounding material. To further understand this phenomenon, V-pit cross sections from the 9% and 15% films were characterised using transmission electron microscopy in combination with energy dispersive X-ray spectroscopy. This identified a subsurface indium-deficient region surrounding the V-pit in the lower indium content film, which was not present in the 15% sample. Although this cannot directly explain the impact of ridges on turn-on voltage, it is likely to be related. Overall, the data presented here demonstrate the potential of PC-AFM in the field of III-nitride semiconductors.

## 1. Introduction

With a direct bandgap which can be tuned from ultraviolet (3.5 eV) to infrared (0.7 eV) [1] with increasing indium fraction, In${}_{x}$Ga${}_{1-x}$N is a semiconducting III-V materials system of great importance to the optoelectronics industry. Commonly referred to as InGaN, its chief application is in light emitting diodes (LEDs) [2,3], in which the active region consists of thin InGaN quantum wells (QWs) separated by pure GaN barriers.

Although the production of GaN single-crystal substrates is possible [4], the extreme cost of the procedure means InGaN structures, such as those in LEDs, are usually grown by metal organic chemical vapour deposition (MOCVD) on “pseudo-substrates” of GaN grown on sapphire (or other mismatched substrates). Unfortunately the large 16% lattice mismatch between GaN and sapphire [5] leads to threading dislocations with a typical density in the pseudo-substrate of $10^{8}$ cm${}^{-2}$ [6], and these propagate into the InGaN layers.

A recent cathodoluminescence study on thick InGaN layers has demonstrated that the core region of such threading dislocations is associated with enhanced light emission [7]; the authors propose this is due to indium concentrating in the dislocation strain field, thereby localising carriers in the vicinity of the dislocation core. This surprising result has implications for the role of defects in InGaN-based light emitters, and warrants further investigation. To this end, techniques which address the electrical properties of materials at the micro/nanoscale can be extremely valuable; various electrical atomic force microscopy (AFM) methods are highly suitable in this regard. However, in past literature these have been more widely applied to GaN rather than InGaN.

For example, conductive-AFM (C-AFM) analysis of pure GaN has demonstrated that certain threading dislocations act as current leakage paths under reverse bias [8,9,10,11], with the density of such dislocations being dependent on the magnitude of negative bias applied [12,13,14]. Law et al. [13] have suggested dislocation current leakage is due to impurities along or defects near dislocations, but the effect is still poorly understood.

C-AFM studies are complicated by the formation of an insulating layer wherever current flow occurs on the sample surface [12,15], with scanning Auger spectroscopy suggesting this layer is gallium oxide created through an electrochemical reaction with atmospheric oxygen [16]. Nonetheless, C-AFM has been found to induce surface changes in GaN even while operating under N_2_ atmosphere [17], and these effects substantially increase the difficulty of obtaining reliable C-AFM results, while also potentially preventing repeat scans of the same area of surface due to reduced current flow.

A recent study by Kim et al. [18] used C-AFM on an InGaN multiple QW structure in an attempt to characterise large V-pits (hexagonal pits which terminate threading dislocations [19]). High current was detected only around the edges of the pits; very similar features have previously been identified by Oliver [20] as artefacts arising from changes in tip-sample contact area. Consequently, current variation around sharp topographic features (on the order of the tip radius) must especially be treated with caution, with Oliver further suggesting that future studies should correlate electrical AFM data with other experimental techniques to help identify artefacts.

The recently developed technique of photoconductive AFM (PC-AFM) builds upon C-AFM by including a one-sun light source incident on the lower surface of a transparent sample, underneath the tip location. Here “one-sun” refers to the light approximating solar illumination, since PC-AFM is commonly used on materials for photovoltaic applications [21]. This gives access to nanoscale photocurrent measurements at the sample surface, allowing us to probe carrier behaviour under illumination. This setup also allows for current-voltage (I-V) curves to be taken at specific locations on the sample surface, by first measuring a topography map and then bringing the tip into stationary contact on a selected point of the surface and varying the voltage while measuring the generated current. (See Section 4 for further description of PC-AFM).

In this study, in addition to providing additional information on the properties of dislocations in thick InGaN layers, one aim was to gauge the usefulness of PC-AFM as an InGaN characterisation technique, highlighting not only its strengths but also some of its challenges and pitfalls. The samples studied were thick (∼130 nm) InGaN films with various indium contents grown on c-plane sapphire. Such thick InGaN layers have potential applications in solar cells [22] (making PC-AFM particularly relevant), while also providing a useful starting point for related studies of QWs for LEDs. Peakforce AFM (PF-AFM) was also used to gain higher resolution topography data and confirm sample surface morphology. Finally, V-pit cross sections were analysed by transmission electron microscopy (TEM) in an attempt to validate and explain PC-AFM observations.

## 2. Results

### 2.1. Samples

The samples studied in this paper were initially characterised by X-ray diffraction (XRD) to determine their bulk indium content, thickness, and relaxation—results are given in Table 1. It is worth noting that the thickness of the In_0.15_Ga_0.85_N layer could not be obtained because the intensity of interference fringes around the analysed XRD peak were too low.

### 2.2. PC-AFM

**Figure 1:**
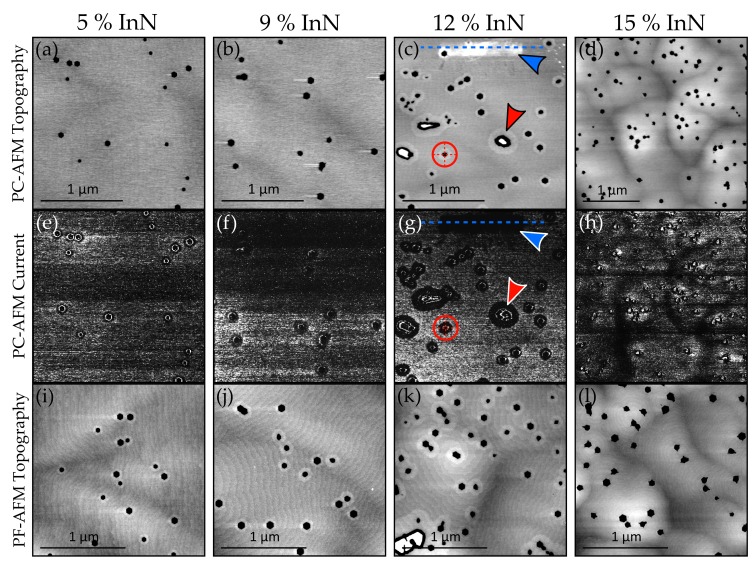
Topography maps obtained from PC-AFM for (**a**) In_0.05_Ga_0.95_N (Δz = 6.5 nm) (**b**) In_0.09_Ga_0.91_N (Δz = 6.0 nm) (**c**) In_0.12_Ga_0.88_N (Δz = 6.0 nm) and (**d**) In_0.15_Ga_0.85_N (Δz = 12.0 nm), along with their corresponding current maps in (**e**) (ΔI = 6.0 pA) (**f**) (ΔI = 30.0 pA) (**g**) (ΔI = 250.0 pA) and (**h**) (ΔI = 30.0 pA), all obtained under illumination. Blue arrows highlight the presence of an insulating layer, while red arrows indicate an example of a defect ridge. Applied bias was +5.5 V for In_0.05_Ga_0.95_N, and +4.5 V for all other samples. PF-AFM topography maps for each sample are given in (**i**) (Δz = 3.5 nm) (**j**) (Δz = 3.0 nm) (**k**) (Δz = 4.5 nm) and (**l**) (Δz = 6.0 nm).

Figure 1 presents the PC-AFM topography and current maps for all samples, along with the surface topography measured by PF-AFM. PC-AFM without illumination resulted in nearly no measurable current flow, so all PC-AFM data in this figure were obtained under illumination. In addition, topography from PC-AFM shows low spatial resolution since contact mode with a higher radius tip was required; PF-AFM was therefore used to obtain higher quality topography images.

**Figure 2:**
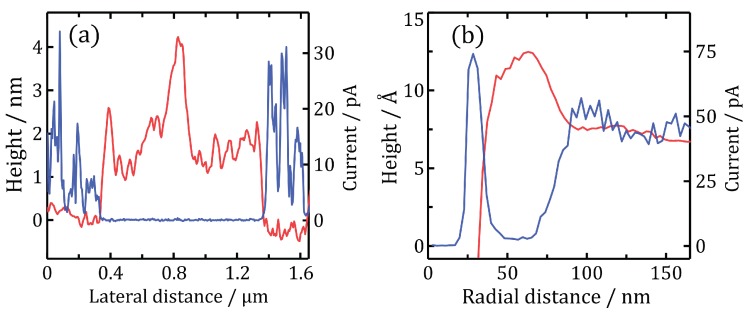
(**a**) Height (red line) and photocurrent (blue line) cross sections of the insulating layer, acquired from blue dashed lines in Figure 1c,g; (**b**) Average radial height (red line) and photocurrent (blue line) cross sections of a V-pit ridge, centred on the V-pit marked by red circles in Figure 1c,g.

Focussing on the PC-AFM topography of the In_0.12_Ga_0.88_N film (Figure 1c), hexagonal V-pits and trench defects are visible, along with elevated ridges around both types of defect (example indicated by the red arrow). PF-AFM confirms these observations (Figure 1k). The raised plateau (blue arrow) corresponds to a layer formed by a previous PC-AFM scan over this smaller area; comparing with the relevant current map in Figure 1g, the layer appears to be insulating. This suggests that oxidation is still occurring at the surface of InGaN as it did for pure GaN—such a layer formed on all InGaN samples during PC-AFM scans. Also noticeable is high current flow around the edges of defects; however, this is very similar to the effect observed by Kim et al. [18] which was likely due to tip-sample contact area changes, and as such should be treated with suspicion. A particularly noteworthy feature is the current drop which occurs around each defect (e.g., see red arrow), corresponding very well to the raised ridges in the topography. The ridges exhibit plateaus up to several 10s of nm wide, and across these flat raised regions the tip-sample contact area is expected to be constant. Figure 2 presents height and photocurrent cross sections of the insulating layer, along with average radial height/current cross sections of a ridge around a typical V-pit. The positions from which the layer and ridge cross sections were taken are indicated in Figure 1c,g by blue dashed lines and red circles respectively.

PC-AFM results gained from the In_0.05_Ga_0.95_N and In_0.09_Ga_0.91_N samples (Figure 1a,b,e,f) are similar to those from the In_0.12_Ga_0.88_N film; however, the ridges around the V-pits cannot be made out in the PC-AFM topography. On the other hand, they are visible in the higher spacial resolution PF-AFM images (Figure 1i,j), and the corresponding photocurrent maps again indicate reduced current flow in these regions. (It is worth noting that the streaks after V-pits in Figure 1b are artefactual, arising from feedback gain being too high during image acquisition).

Given that the photocurrent drops cannot be directly correlated with the ridges in this case, it was necessary to analyse the measured photocurrent drop width distribution obtained from PC-AFM and compare it to the ridge width distribution from PF-AFM topography. These distributions were found by taking the average radial height cross section around 54 pits in PF-AFM, and the average radial current cross section of 32 dislocations in the PC-AFM current results.

The measured distributions from this analysis are presented in Table 2; for both samples, the width distributions of the ridges and the current drops match well. This strongly suggests that the photocurrent drops still correspond to the ridges around each defect for the In_0.05_Ga_0.95_N and In_0.09_Ga_0.91_N samples. PF-AFM was also used to extract average ridge height from 180 V-pits across all three low indium content samples, with the result of 3.33 ± 0.05 Å—i.e., on the order of one atomic monolayer.

Finally, it’s also worth noting the lower current measured in regions with no pits in both Figure 1e,f. We speculate that this is due to the tip becoming contaminated and leading to a contact resistance increase, before sudden height changes at a V-pit place more force on the tip and clear some of the contamination, causing a current increase on the first line after encountering a V-pit.

Results from the In_0.15_Ga_0.85_N sample (Figure 1d,h,l) make it clear that this film is substantially different from those previously analysed. The surface is significantly rougher, with noticeable hills and valleys over a 12 nm height scale. Ridges are not visible in either the PC-AFM or PF-AFM topography, and no photocurrent drops are observed around the pits in the current map. The current map also shows connected regions of low current which correspond very well to the valleys observed in the topography. Current readings within the pits are different from before, with one side of the pit exhibiting higher current than the other side. This is likely due to the feedback system not keeping up with rapid height changes, leading to lower contact force on one side (and hence lower current) and higher contact force on the other side (increased current). This effect may be present only in the In_0.15_Ga_0.85_N film results due to different feedback gains or a differently shaped tip compared to similar measurements on the other samples. It is worth noting that to facilitate observation of the features associated with the valleys between the dislocations, Figure 1d,h are presented at a different lateral scale to the other parts of the figure.

**Figure 3:**
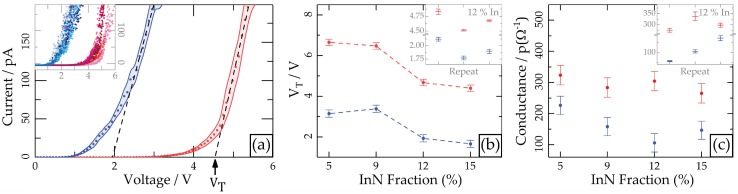
Current-voltage data obtained under illumination (blue) and no illumination (red) away from any defects. (**a**) Inset: raw data from In_0.12_Ga_0.88_N showing twenty I-V curves, ten with no illumination and ten with illumination. Main: average curves, which have been linearly fitted to extract a “turn-on” voltage ($V_{T}$, x-intercept) and conductance (*G*, gradient); (**b**) $V_{T}$ for all InGaN thick films, where the error bars represent the standard deviation in repeated measurements over the course of the session. Inset: repeatedly measured $V_{T}$ for the In_0.12_Ga_0.88_N film, with error bars representing the standard error in the x-intercept of the linear fit; (**c**) Conductance results presented in a similar manner to (**b**).

I-V curves obtained away from any defects exhibit diode-like behaviour, likely because the tip forms a rectifying contact with the surface. They are also quite noisy (see Figure 3a inset), so analysing them individually would lead to significant error. Instead, an effective method is to take ten I-V curves in the region of interest with the same sampling rate and voltage range, and then average the measured current at each voltage point over all the I-V curves. As such the standard error in each current value can also be found, which allows for more meaningful comparison of current-voltage behaviour on different samples with/without illumination. The linear portion of the curve can then be fitted with a straight line, and extrapolated back to obtain a “turn-on” voltage, $V_{T}$; the gradient of this line also gives an indication of the conductance of the system, *G*. All of the above leads to quite a cluttered I-V plot, so to simplify interpretation error bars are represented by lines; the final result can be seen in Figure 3a. Together, $V_{T}$ and *G* provide a means of quantitatively comparing the current-voltage characteristics of different samples away from any V-pit ridges, as shown in Figure 3b,c.

All films were analysed in one session with the same AFM tip, in the testing order In_0.12_Ga_0.88_N → In_0.09_Ga_0.91_N → In_0.05_Ga_0.95_N → In_0.12_Ga_0.88_N → In_0.15_Ga_0.85_N → In_0.12_Ga_0.88_N. The In_0.12_Ga_0.88_N film was repeated throughout to track the tip status; any change in $V_{T}$ or *G* for this sample between the different measurements would indicate a change in the tip. The inset of Figure 3b shows how $V_{T}$ for the In_0.12_Ga_0.88_N film varied over the course of the session, with the error bars corresponding to the standard error of extrapolating the linear fit to $V_{T}$ in each case. It can be seen there is no overall drift in $V_{T}$ as the test is repeated, but the standard error of extrapolation is much lower than the change in $V_{T}$ each time, which indicates that tip changes dominate the error in these measurements. As such the standard deviation of these three measurements is used to roughly gauge the error in all the measurements, and is plotted as error bars in the main plot of Figure 3b. The value for the In_0.12_Ga_0.88_N film in the main plot is the average of the three repeated measurements.

$V_{T}$ was always much lower when light was incident on the film; this is expected, since incoming photons will generate electron-hole pairs in the InGaN, increasing carrier density at the Schottky contact with the PC-AFM tip and thereby reducing the Schottky barrier height. Hence the applied voltage required to overcome the Schottky barrier ($V_{T}$) is lower under illumination. The trend between different samples is less clear; generally, $V_{T}$ seems to decrease with increasing In content, which could be expected from the corresponding decrease in bandgap.

Figure 3c shows data for conductance, *G*, obtained from the same experiment; in this case the variance of the repeat measurements is large (inset), so no comparison can clearly be made between samples. Carrying out more repeats would be difficult due to the trade-off between identifying tip changes and further damaging the tip to do so. Interestingly, *G* was always lower when the light was on, which is unexpected. This could be explained by the insulating layer that builds up on the surface once current starts to flow; higher initial current may lead to more insulating layer being generated, which would reduce the measured conductance. Conversely, $V_{T}$ should be unaffected since no current has been generated at this point in the I-V curve and an electrochemical reaction should not be able to occur without the movement of charge.

**Figure 4:**
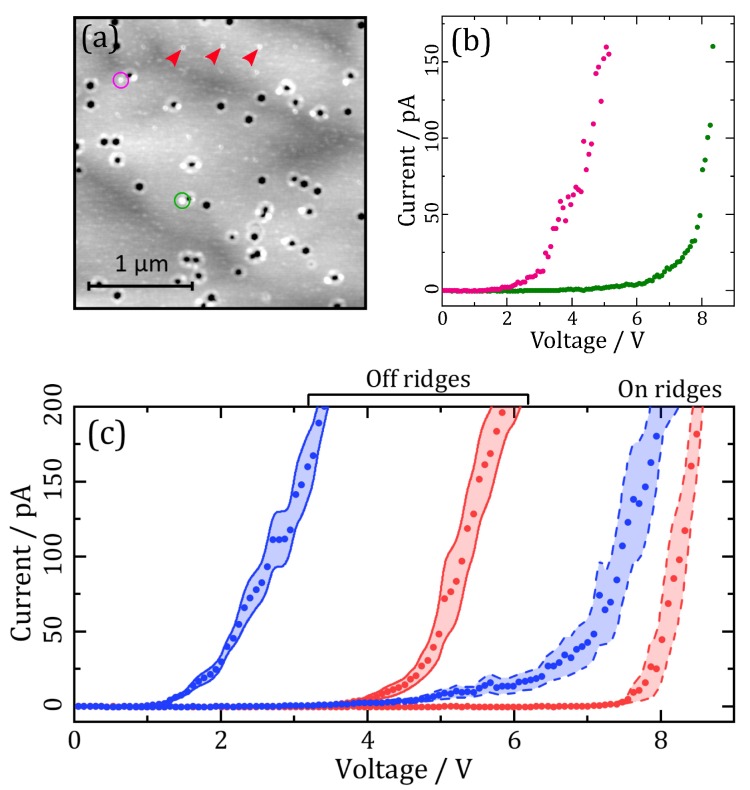
(**a**) Topography map taken after I-V curves were measured on the In_0.12_Ga_0.88_N layer. The pink and green circles enclose a spot of insulating layer where an I-V curve was previously taken; (**b**) I-V curves obtained with illumination from the green and pink enclosed regions in (**a**); (**c**) Overall average results for In_0.12_Ga_0.88_N, under illumination (blue) and no illumination (red), on and away from dislocation ridges.

Measuring I-V curves also provides a way to quantify nanoscale features such as the defect ridges, since the location at which the I-V curve is taken can be specified. A key consideration is that the ridges are not much larger than the tip radius, so any drift in the sample after the initial topography map is taken could lead to the tip missing the ridge and mistakenly taking an I-V curve of background material. Figure 4a,b illustrate how the formation of insulating layer can actually be helpful in identifying when this occurs, allowing for the resultant curves to be excluded from the analysis. The topography map taken after I-V measurements (with the light on) in Figure 4a shows that if the tip misses the ridge, the insulating layer also forms away from the ridge (pink circle). On the other hand, if the tip correctly contacts the ridge, we see insulating layer there (green circle). Figure 4b shows the individual I-V curves obtained from the pink and green circle measurements—there is a large difference between the two. Hence the “pink” result should be excluded from our ridge current-voltage analysis. Figure 4a was acquired after extensive use of the AFM tip, so the light “speckles” over the surface can be attributed to tip damage. However, these artefactual speckles must not be confused with insulating regions which were formed in regular arrays on the surface by I-V curve measurements (e.g., red arrows).

Figure 4c presents the average I-V curves obtained with the light on and off, away from defect ridges and on them, using this method on the In_0.12_Ga_0.88_N layer. Measured turn-on voltage was much higher on the ridges; the increases were 4.93±0.07 V and 3.2±0.04 V with the light on and off respectively. Interestingly, conductance was also higher on the ridges once current did start flowing, with an increase of 34±11 p(Ω${}^{-1}$) and 130±15 p(Ω${}^{-1}$) for the light on and off; but as previously stated, conductance measurements are made unreliable due to the insulating layer.

### 2.3. TEM

**Figure 5:**
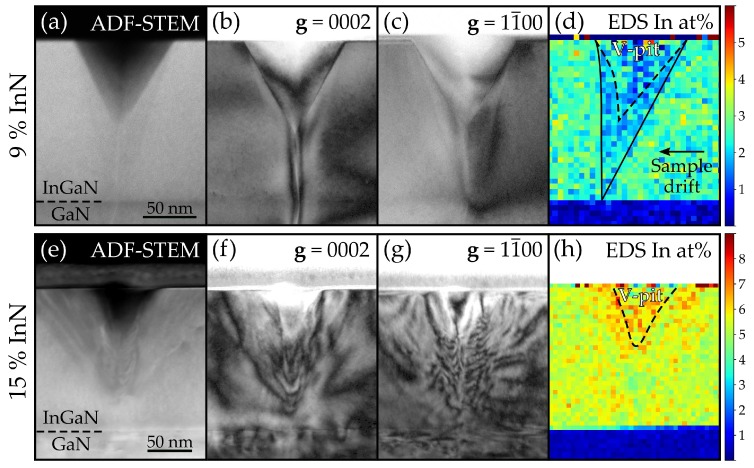
TEM results for a V-pit cross section in the In_0.09_Ga_0.91_N sample showing (**a**) ADF-STEM (**b**) bright field image taken with **g** = 0002 (**c**) bright field image taken with **g** = $1\bar{1}00$ and (**d**) STEM-EDS indium concentration map - the V-pit edges are indicated by a dashed line, and the solid line marks the boundary of a low indium region. (**e**–**h**) show similar results for a V-pit cross section in the In_0.15_Ga_0.85_N sample. All images taken looking down the $\langle 11\bar{2}0\rangle$ zone axis.

Figure 5 presents TEM results for the In_0.09_Ga_0.91_N and In_0.15_Ga_0.85_N layers, focussing on a single V-pit cross-section in each. Comparison between these two samples is particularly apt since In_0.09_Ga_0.91_N is representative of the samples with ridges around pits and photocurrent drops (low indium content samples), while In_0.15_Ga_0.85_N had no ridges or drops.

Looking at the annular dark field (ADF) scanning TEM (STEM) image of the In_0.09_Ga_0.91_N film in Figure 5a, there is clear contrast between the pure GaN buffer layer and the InGaN thick layer, with the GaN appearing darker due to its lower average atomic mass. Of most interest is the faintly visible darker region around the V-pit extending down to the GaN interface, which suggests lower indium concentration in this area. This is confirmed by the STEM energy-dispersive X-ray spectroscopy (EDS) map of indium concentration (Figure 5d), once the drift in the specimen has been accounted for. Figure 5b,c present bright field TEM images with **g** = 0002 and $1\bar{1}00$ respectively, and demonstrate that the only dislocation present is the threading dislocation at the V-pit apex. Faint contrast is also visible at the boundary of the indium-deficient region.

Figure 5e–h present results from the same methods applied to the In_0.15_Ga_0.85_N V-pit. Focussing on Figure 5e, ADF-STEM no longer shows clear contrast between the GaN buffer layer and the InGaN, suggesting strain is affecting the contrast. More interestingly, the bright field TEM images suggest there are multiple dislocations branching out around the V-pit. STEM-EDS shows no reduction in indium concentration around the V-pit, and perhaps even indicates a slightly increased concentration close to the facets.

The STEM-EDS maps provide indium content as atomic percent rather than as *x* in In${}_{x}$Ga${}_{1-x}$N. Hence, the values on the scale bars in Figure 5 should be doubled to allow comparison with the XRD results in Table 1. Such a comparison, however, suggests a lower average indium content in the STEM-EDS measurement than in XRD. This is likely due to a systematic error in the EDS quantification method, but does not effect the above observations concerning relative indium contents.

## 3. Discussion

A key observation in this study has been how surface current flow is altered by elevated ridges around defects in the 5–12% InN samples; illuminated PC-AFM maps show that much less current is measured on these ridges than on the rest of the surface (Figure 1). In addition, $V_{T}$ was seen in Figure 3 to decrease with increasing In content and was also found to be much greater on V-pit ridges (Figure 4).

These data are consistent with the STEM-EDS results in Figure 5d, which clearly show an indium-deficient region around the V-pit facets in the In_0.09_Ga_0.91_N film. Hence the low current flow and higher $V_{T}$ in the ridge area is at least partly attributable to the decreased In content in this region; however, it should be noted that the position of the ridge and the indium-deficient zone do not exactly overlap. To understand how this sharp-edged low indium region seen in EDS could have formed, the growth mechanism and morphology of the V-pit must be appreciated.

V-pits within MOCVD-grown QW structures have been analysed extensively in previous literature [19,23,24,25,26,27], and likely arise from a similar growth process to the V-pits in this study’s thick InGaN layers. Nucleation occurs due to strain relaxation; hence in QW structures it usually initiates during first QW growth [25] due to the lattice mismatch between GaN and InGaN–similarly it nucleates at the lower GaN/InGaN interface in our In_0.09_Ga_0.91_N sample. From this point the V-pit growth occurs due to slower growth rate on the exposed $\left\{10\bar{1}1\right\}$ planes in comparison with the upper surface (0001) plane; once layer growth is terminated, an open V-pit is left at the surface.

Applying this growth mechanism to our thick layers, lower indium incorporation on the facets of the V-pit as it grows could directly explain the shape of the indium-deficient region in ADF-STEM. Hence the boundary plane around the low indium area is determined by the relative growth rate in the V-pit compared to the rate on the adjacent (0001) plane. This hypothesis is reinforced by the fact that V-pit sidewall QWs in multiple QW structures also have lower indium content than (0001) QWs [28], providing evidence that lower incorporation of indium on V-pit facets during growth does indeed occur. These indium-deficient regions generate subsurface wider bandgap material surrounding the V-defects, which would act as a barrier to carrier diffusion, leading to a similar dislocation self-screening effect as described by Hangleiter et al. [23].

TEM results on the In_0.15_Ga_0.85_N film can also help explain the lack of ridges and photocurrent drops in the PC-AFM results from the same sample. V-pit morphology in this film is significantly different from the In_0.09_Ga_0.91_N film, with no low indium region and many dislocations branching around the pit. Similar dislocation branching has been observed before in MOCVD-grown high indium content thick InGaN films [29,30]. In particular, Ponce et al. [29] analysed two thick layers with 10% and 15% InN fraction respectively, only finding branching dislocations in the latter; this agrees well with this paper’s observation. The differences in pit morphologies between In_0.09_Ga_0.91_N and In_0.15_Ga_0.85_N are likely related to the lack of ridges in the In_0.15_Ga_0.85_N layer.

## 4. Materials and Methods

All samples were grown on c-plane sapphire substrates by MOCVD in a Thomas Swan 6 × 2 in. close-coupled showerhead reactor; the exact method is described in more detail elsewhere [7]. All are doped with Si to ca. $5\times 10^{18}$ cm${}^{-3}$, and rest on about 5 μm of GaN (of which 2 μm is undoped and 3 μm Si-doped to $5\times 10^{18}$ cm${}^{-3}$). XRD analysis of these films was performed in a Philips X’Pert diffractometer using Cu Kα_1_ radiation with a double-bounce Ge (220) asymmetric monochromator and a double-bounce analyser.

**Figure 6:**
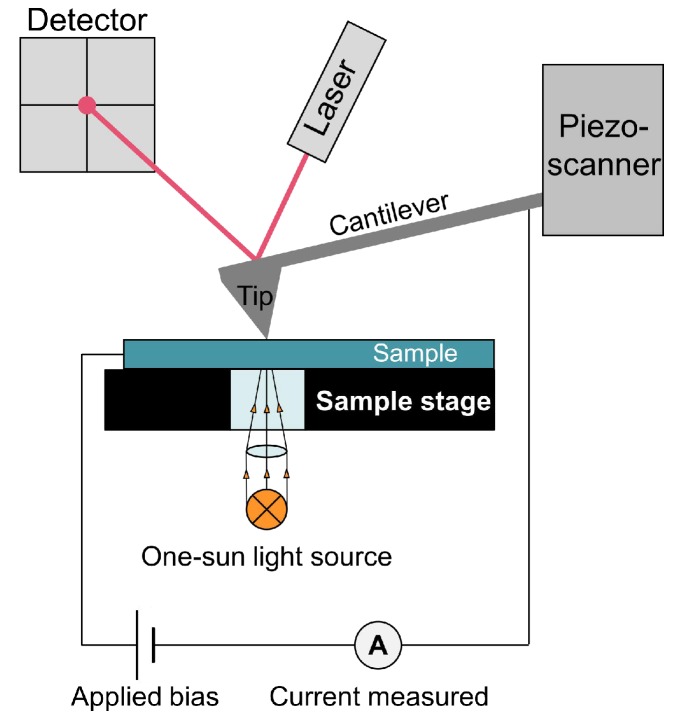
Simple schematic of the PC-AFM setup.

Figure 6 displays a simple schematic of the PC-AFM instrumentation; the setup is very similar to usual C-AFM, but includes a 75 W ozone free xenon lamp (wavelength range 200–2500 nm) which simulates solar illumination. AFM maps were acquired using a Bruker Dimension Icon AFM with an applied bias range of −10 V to +10 V. Contact mode with a Pt/Ir coated Sb (n) doped Si tip was used for PC-AFM, while PF-AFM was carried out with a sharp nonconductive Si${}_{3}$N${}_{4}$ tip. Obtaining I-V curves required a Si tip coated with highly conductive boron-doped diamond, to maintain tip conductivity over repeated measurements. The InGaN films were electrically contacted to the sample stage using silver paint and conductive clips. AFM topography data processing consisted of removal of second order polynomial background and median line matching.

TEM samples were prepared using standard mechanical polishing followed by Ar${}^{+}$ ion milling, and all imaging was performed using an FEI TecnaiOsiris microscope at a beam energy of 200 keV. The STEM-EDS map was quantified using the Cliff-Lorimer method [31].

## 5. Conclusions

In summary, the main observation was consistently lower current measured on ridges around defects in the 5–12% InN samples, with TEM suggesting this effect is related to sub-surface indium-deficient regions present around V-pits, which form due to lower indium incorporation on pit facets during growth.

The high indium content film (In_0.15_Ga_0.85_N) was found to be significantly different from the others, with no ridges and lower measured current in the surface valleys. TEM analysis revealed that this sample’s V-pit morphology was also substantially different, with extra branching dislocations and no indium-deficient zone, which may explain why no ridges are present around the pits.

We can also comment on the applicability of PC-AFM to further characterise the InGaN materials system. Illumination allowed for current maps to be obtained more easily at a lower applied bias, since the turn-on voltage significantly decreased with light exposure. In general, PC-AFM suffers from issues such as tip inconsistency and induced sample surface changes, but its ability to observe new phenomena in a III-nitride semiconductor system has been demonstrated in this study.

## Figures and Tables

**Table 1:** List of InGaN sample properties, gained from XRD analysis of the symmetric 0002 reflection.

Sample	InN fraction/%	Thickness/nm	Relaxation/%
In_0.05_Ga_0.95_N	4.9±0.5	129±2	2±3
In_0.09_Ga_0.91_N	8.6±0.5	136±2	−2±3
In_0.12_Ga_0.88_N	12.4±0.5	131±2	5±2
In_0.15_Ga_0.85_N	14.7±0.5	-	7±4

**Table 2:** Mean ridge widths (${\bar{W}}_{r}$) and photocurrent drop widths (${\bar{W}}_{PC}$) with corresponding standard deviations (σ) obtained from analysing PF-AFM topography and PC-AFM current maps of In_0.05_Ga_0.95_N and In_0.09_Ga_0.91_N.

Sample	PF-AFM/nm	PC-AFM/nm
In_0.05_Ga_0.95_N	${\bar{W}}_{r}=37$, σ=6	${\bar{W}}_{PC}=37$, σ=6
In_0.09_Ga_0.91_N	${\bar{W}}_{r}=46$, σ=5	${\bar{W}}_{PC}=46$, σ=10
